# Antitumor Activity of the Xanthonoside XGAc in Triple-Negative Breast, Ovarian and Pancreatic Cancer by Inhibiting DNA Repair

**DOI:** 10.3390/cancers15245718

**Published:** 2023-12-06

**Authors:** Juliana Calheiros, Liliana Raimundo, João Morais, Ana Catarina Matos, Sonia Anna Minuzzo, Stefano Indraccolo, Emília Sousa, Marta Correia da Silva, Lucília Saraiva

**Affiliations:** 1 LAQV/REQUIMTE, Laboratório de Microbiologia, Departamento de Ciências Biológicas, Faculdade de Farmácia da Universidade do Porto, 4050-313 Porto, Portugal; julianameixedo@ua.pt (J.C.); liliana-raimundo@live.com (L.R.); up201805193@edu.fc.up.pt (J.M.); catarina.matos99@hotmail.com (A.C.M.); 2 Department of Surgery Oncology and Gastroenterology, University of Padova, 35128 Padova, Italy; soniaanna.minuzzo@unipd.it (S.A.M.); stefano.indraccolo@unipd.it (S.I.); 3 Veneto Institute of Oncology IOV—IRCCS, 35128 Padova, Italy; 4 Laboratório de Química Orgânica e Farmacêutica, Departamento de Ciências Químicas, Faculdade de Farmácia da Universidade do Porto, 4050-313 Porto, Portugal; esousa@ff.up.pt (E.S.); m_correiadasilva@ff.up.pt (M.C.d.S.); 5 CIIMAR—Centro Interdisciplinar de Investigação Marinha e Ambiental, Terminal de Cruzeiros do Porto de Leixôes, 4450-208 Matosinhos, Portugal

**Keywords:** xanthone derivative, anticancer agent, DNA damage response, PARPi

## Abstract

**Simple Summary:**

DNA repair inhibition constitutes a promising anticancer strategy, particularly in triple-negative breast cancer (TNBC), ovarian cancer and pancreatic ductal adenocarcinoma (PDAC). XGAc is a xanthonoside previously described as a potent cancer cell growth inhibitor. Herein, we aimed to evaluate the antitumor activity of XGAc in TNBC, ovarian cancer and PDAC cells, either alone or in combination with the poly(ADP-ribose) polymerase inhibitor (PARPi) olaparib. XGAc exhibits antiproliferative activity in TNBC, ovarian cancer and PDAC cells, also proving to be effective against patient-derived ovarian cancer cells and drug-resistant cancer cells. XGAc inhibited cancer cell migration, induced apoptosis and S-phase cell cycle arrest, and triggered genotoxicity by inhibiting the expression of homologous recombination DNA repair proteins in TNBC, ovarian cancer and PDAC cells. Importantly, XGAc displayed synergistic effects with olaparib, demonstrating its potential in combination therapy. Altogether, XGAc reveals itself to be a valuable anticancer agent for hard-to-treat cancers.

**Abstract:**

Dysregulation of the DNA damage response may contribute to the sensitization of cancer cells to DNA-targeting agents by impelling cell death. In fact, the inhibition of the DNA repair pathway is considered a promising anticancer therapeutic strategy, particularly in combination with standard-of-care agents. The xanthonoside XGAc was previously described as a potent inhibitor of cancer cell growth. Herein, we explored its antitumor activity against triple-negative breast cancer (TNBC), ovarian cancer and pancreatic ductal adenocarcinoma (PDAC) cells as a single agent and in combination with the poly(ADP-ribose) polymerase inhibitor (PARPi) olaparib. We demonstrated that XGAc inhibited the growth of TNBC, ovarian and PDAC cells by inducing cell cycle arrest and apoptosis. XGAc also induced genotoxicity, inhibiting the expression of DNA repair proteins particularly involved in homologous recombination, including BRCA1, BRCA2 and RAD51. Moreover, it displayed potent synergistic effects with olaparib in TNBC, ovarian cancer and PDAC cells. Importantly, this growth inhibitory activity of XGAc was further reinforced in a TNBC spheroid model and in patient-derived ovarian cancer cells. Also, drug-resistant cancer cells showed no cross-resistance to XGAc. Additionally, the ability of XGAc to prevent cancer cell migration was evidenced in TNBC, ovarian cancer and PDAC cells. Altogether, these results highlight the great potential of acetylated xanthonosides such as XGAc as promising anticancer agents against hard-to-treat cancers.

## 1. Introduction

Triple-negative breast cancer (TNBC), advanced ovarian cancer and pancreatic ductal adenocarcinoma (PDAC) are known for their high therapeutic resistance [1]. These cancers commonly exhibit pathogenic variants in homologous recombination DNA repair genes, particularly *BRCA*, *ATM*, *BARD1*, *RAD51* and *PALB2*, resulting in impaired DNA double-strand breaks (DSBs) repair [2,3]. Since homologous recombination is an essential DNA repair pathway to preserve genomic stability, its inhibition, namely, by targeting ATM, ATR, CHK1, CHK2, WEE1, RAD51 and RAD52, represents a promising therapeutic strategy in cancer treatment. These inhibitors are currently under study and some of them have reached clinical trials, supporting further studies aiming to unravel new homologous recombination inhibitors [4].

BRCAs are critical components of homologous recombination DNA repair. Mutations in these proteins are related to increased cancer risk [5]. Despite this, compiled data have highlighted that cancer patients harbouring BRCA mutations (mutBRCA) exhibit increased sensitivity to platinum-based chemotherapy and poly(ADP-ribose) polymerase (PARP) inhibitors (PARPis), demonstrating that BRCA deficiency may improve therapeutic response [6]. In fact, PARPis have emerged as an encouraging targeted therapy for mutBRCA-associated tumors, since the mutual disfunction of BRCA and PARP (an essential enzyme in DNA single-strand break repair) can induce synthetic lethality in DNA repair defective cancer cells [3]. Briefly, PARPis trap PARP enzymes at the damage site by binding to the ADP ribosyltransferase catalytic domain, promoting the progression of single-strand breaks to DSBs, impairing the progression of replication forks and requiring a functional homologous recombination DNA repair to overcome this inhibition [5]. The PARPi olaparib was approved for mutBRCA locally advanced or metastatic breast cancer, as maintenance therapy of mutBRCA advanced ovarian cancer and for metastatic PDAC [7]. However, although effective in mutBRCA-associated DNA repair defective cancers and in an additional subset of cancer patients exhibiting BRCAness phenotype (harbouring DNA repair defects in non-*BRCA* genes, producing BRCA-like homologous recombination impairment), most cancers harbour wild-type BRCA (wtBRCA), which greatly limits the clinical potential of PARPis as a monotherapy [4]. Since the percentage of cancer patients responsive to PARPis is very limited, studies have been focused on establishing combination strategies to widen the application of PARPis to a larger population of cancer patients. In fact, PARPis have shown synergistic effects in combination with several cytotoxic agents, highlighting their promising contribution to the effectiveness of the standard-of-care therapies [5]. However, acquired resistance to PARPis has been commonly observed, particularly by residual DNA repair activity, involving restoration or overexpression of DNA repair machinery [3]. For instance, an increased RAD51 foci formation is commonly observed in mutBRCA cancers and its overexpression is associated with PARPi resistance in breast cancer cells [8,9]. Another strategy for extending the spectrum of PARPi applications is their combinatorial use with BRCAness inducers [4].

Xanthones are *O*-heteroaromatic tricyclic scaffolds, which provide a wide range of derivatives with several biological responses, representing a privileged structure for anticancer drug development [10]. Particularly, this class of oxygen-containing heterocyclic compounds can intercalate into the base group pairs of DNA due to the appropriate planarity of the xanthone ring, causing DNA damage in cancer cells through non-covalent interaction with DNA [11,12]. In addition, the glycosidic moiety of natural glycosides of flavonoids and xanthones may exhibit biological activities that positively affect the antitumor activity [13]. Furthermore, the acylation process proves to be important to the cell growth inhibitory activity, also improving the cell membrane penetration of compounds such as flavonoids [14]. Thus, acetylated xanthone glycosides are revealed to be promising inhibitors of cancer cell growth.

**Figure 1:**
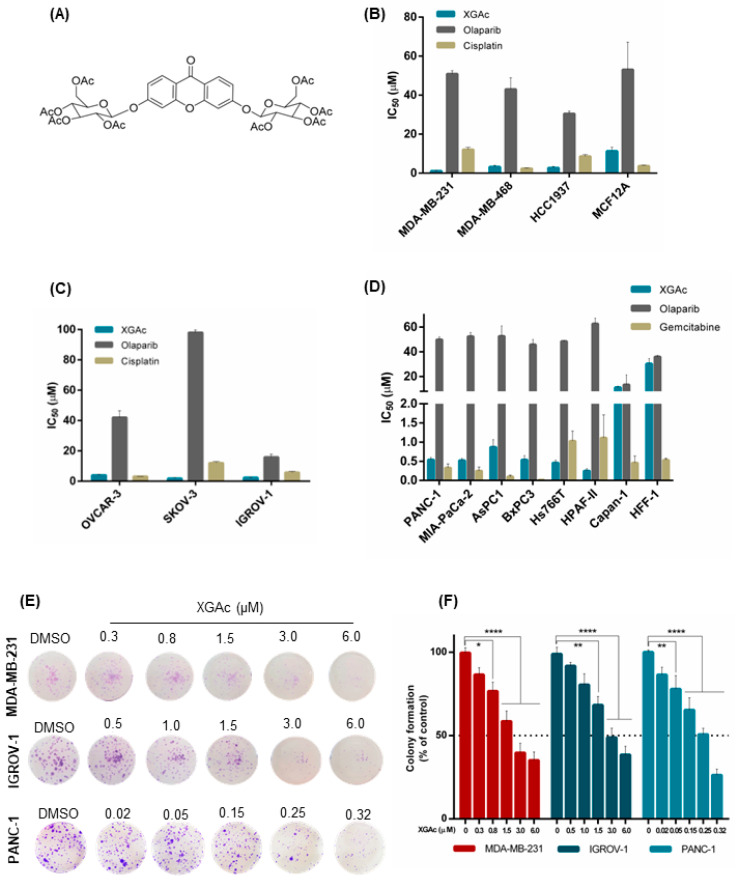
Growth inhibitory activity of XGAc in TNBC, ovarian cancer and PDAC cells. (**A**) XGAc chemical structure. (**B**–**D**) IC_50_ values of XGAc, olaparib, cisplatin and gemcitabine in TNBC (**B**), ovarian cancer (**C**) and PDAC (**D**) cells were determined after 48 h of treatment by SRB assay; data are mean ± SEM of three independent experiments (two replicates each). (**E**,**F**) Effect of XGAc on colony formation of cancer cells after 15 days of treatment. In (**E**), representative experiments are shown. In (**F**), quantification of colony formation; growth obtained with DMSO was set as 100%; dotted lines represent the 50% growth inhibition. Data are mean ± SEM of three independent experiments; values significantly different from DMSO: * *p* < 0.05, ** *p* < 0.01, **** *p* < 0.0001 (two-way ANOVA followed by Dunnett’s test).

Previously, we disclosed a new acetylated xanthonoside, 3,6-bis(2,3,4,6-tetra-*O*-acetyl-β-glucopyranosyl)xanthone (XGAc; Figure 1A), which inhibited the growth of glioma, melanoma, breast adenocarcinoma, glioblastoma astrocytoma, and non-small cell lung cancer cells [15]. Interestingly, the non-acetylated xanthonoside did not reach 50% of cell growth inhibition, evidencing the relevance of the acylation process to improve the antitumor activity [15]. Herein, we aimed to explore the antitumor activity of XGAc, either as a single agent or in combination with olaparib, in hard-to-treat cancers, including TNBC, ovarian cancer and PDAC.

## 2. Materials and Methods

### 2.1. Compounds

XGAc was synthesized as described in [15]. XGAc, olaparib (AZD2281; Santa Cruz Biotechnologies, Frilabo, Portugal) and gemcitabine (Sigma-Aldrich, Sintra, Portugal) were dissolved in DMSO (Sigma-Aldrich, Sintra, Portugal), while cisplatin (Enzo Life Science, Taper, Sintra, Portugal) was dissolved in saline. The solvent (maximum 0.1%) was included as control.

### 2.2. Human Cancer Cell Lines and Culture Conditions

The human cancer cell lines MDA-MB-231 (triple-negative metastatic breast adenocarcinoma), MDA-MB-468 (triple-negative metastatic breast adenocarcinoma), HCC1937 (triple-negative breast primary ductal carcinoma), OVCAR-3 (metastatic high-grade ovarian serous adenocarcinoma), SKOV-3 (metastatic ovarian serous cystadenocarcinoma), IGROV-1 (ovarian endometrioid adenocarcinoma), AsPC-1 (metastatic pancreatic ductal adenocarcinoma) and Hs766T (metastatic pancreatic adenocarcinoma) were grown in RPMI-1640 medium with UltraGlutamine (Biowest, VWR, Carnaxide, Portugal) supplemented with 10% heat-inactivated fetal bovine serum (FBS, Biowest, VWR, Carnaxide, Portugal), while PANC-1 (pancreatic ductal adenocarcinoma), MIA-PaCa-2 and GEM-resistant MIA-PaCa-2 (generated and kindly supplied by Dr Luigi Sapio from [16]) (pancreatic ductal adenocarcinoma), BxPC3 (pancreatic ductal adenocarcinoma), and HPAF-II (metastatic pancreatic ductal adenocarcinoma) were grown in DMEM high glucose (4.5 g/L glucose) with stable L-glutamine and sodium pyruvate supplemented with 10% FBS. Capan-1 cell line (metastatic pancreatic adenocarcinoma) was cultured in Iscove Modified Dulbecco Media (IMDM) 20% FBS. The normal human foreskin fibroblast HFF-1 cells were grown in DMEM F12 10% FBS. The normal human breast MCF-12A cells were cultured in DMEM F12 10% FBS, 20 ng/mL EGF, 100 ng/mL cholera toxin, 0.01 mg/mL insulin and 500 ng/mL hydrocortisone.

Cells were grown at 37 °C in a 5% CO_2_ humidified atmosphere. Cell number and viability were assessed with trypan blue exclusion assay. Cells were routinely tested for mycoplasma infection using the MycoAlert™ PLUS mycoplasma detection kit (Lonza). Additional information about cells can be found in Supplementary Table S1.

#### Patient-Derived Ovarian Cancer Cells

The patient-derived ovarian cancer (PD-OVCA) cells 1, 9, 41, 49 and 62 were obtained from ascitic effusions of epithelial ovarian cancer patients. These cells were routinely maintained as serial xenotransplants, as previously described [17]. The study was approved by IOV Institutional Review Board and Ethics Committee (EM 23/2017) and performed in accordance with the Declaration of Helsinki. Informed consent was obtained from patients who entered this study. Procedures in animals were conformed to institutional guidelines that comply with national and international laws and policies (EEC Council Directive 86/609, OJ L 358, 12 December 1987) and were authorized by the Italian Ministry of Health (Authorization No. 617/2016 PR). Animal studies are reported in compliance with the ARRIVE guidelines [18].

Patient-derived cells were cultured in RPMI-1640 media with UltraGlutamine (Invitrogen, Milan, Italy) supplemented with 10% FBS (Gibco, Invitrogen), 2 mM L-glutamine, sodium pyruvate, 10 nmol/L HEPES and 100 U/mL Penicillin/Streptomycin, for a maximum of 2 weeks, as in [19]. Additional information about patient-derived cancer cells can be found in Supplementary Table S2.

### 2.3. Cell Viability and Proliferation Assays

The human cell lines MDA-MB-231, MDA-MB-468, HCC1937, MCF12A, OVCAR-3, SKOV-3, IGROV-1, PANC-1, MIA-PaCa-2, GEM-resistant MIA-PaCa-2, AsPC-1, BxPC3, HS766T, HPAF-II, Capan-1 (5.0 × 10^3^) and HFF-1 (1.0 × 10^4^) cells/well were seeded in 96-well plates and allowed to adhere overnight, followed by treatment with serial compound dilutions, for 48 h. Half-maximal inhibitory concentration (IC_50_) values were determined for each cell line by sulforhodamine B (SRB) assay, as described [19].

For PDOVCA 1, 9, 41, 49 and 62 cells, 7.5 × 10^3^ cells/well were seeded in 96-well plates, and the IC_50_ values were determined with the CellTiter96^®^ Aqueous one solution cell proliferation assay (MTS assay; Promega, Italy) after 48 h of treatment, as described [19].

IC_50_ values were determined using the GraphPad Prism software, Version 7.0 (RRID:SCR_002798, La Jolla, CA, USA). Concentration-response curves of XGAc can be found in Supplementary Materials (Figures S1 and S2).

For the colony formation assay, MDA-MB-231, IGROV-1 and PANC-1 (1 × 10^3^) cells/well were seeded in six-well plates and treated at the seeding time with a range of concentrations of XGAc for 15 days. Colonies were fixed with 10% methanol and 10% acetic acid for 10 min and stained with 0.5% crystal violet (Sigma-Aldrich) in 1:1 methanol/H_2_O for 15 min. Colonies containing more than 20 cells were counted.

### 2.4. Mammosphere Generation

HCC1937 cells, 1.5 × 10^3^ cells/well, were seeded in 24-well plates coated with 1% agarose in DMEM F12 supplemented with 20 ng/mL bFGF, 40 ng/mL EGF (Bio-techne, Citomed Lda, Lisboa, Portugal), 1x B27 (Life Technologies, Porto, Portugal), 10 μg/mL insulin (Sigma-Aldrich, Sintra, Portugal) and 2 mM L-glutamine (Sigma) and treated with XGAc at the seeding time, as in [19]. After 72 h, mammosphere growth was monitored using an inverted Nikon TE 2000-U microscope at 100× magnification, with a DXM1200F digital camera and NIS-Elements microscope imaging software (RRID:SCR_014329, Nikon Instruments Inc, Izasa, Carnaxide, Portugal). Spheroid area was quantified using Fiji Software (RRID:SCR_002285) [20].

### 2.5. Cell Cycle and Apoptosis Analysis

MDA-MB-231, IGROV-1 and PANC-1 (1.5 × 10^5^ cells/well) were seeded in six-well plates and allowed to adhere overnight, followed by treatment with XGAc for 48 h. Briefly, cells were stained with propidium iodide (Sigma-Aldrich) and analyzed by flow cytometry for the identification and quantification of cell cycle phases. For apoptosis analysis, cells were stained using the Annexin V-FITC Apoptosis Detection Kit I from BD Biosciences (Enzifarma, Porto, Portugal), according to the manufacturer’s instructions. The Accuri^TM^ C6 flow cytometer, BD Accuri C6 software (RRID:SCR_014422, BD Biosciences) and FlowJo X 10.0.7 software (RRID:SCR_008520, Treestar, Ashland, OR, USA) were used.

### 2.6. Western Blot Analysis

MDA-MB-231, IGROV-1 and PANC-1 (1.5 × 10^5^ cells/well) were seeded in six-well plates, allowed to adhere overnight and treated with XGAc for 48 h. Briefly, protein lysates were quantified using Pierce^®^ BCA Protein Assay reagents (Thermo Fisher Scientific, Porto Salvo, Portugal), resolved by sodium dodecyl sulfate-polyacrylamide gel electrophoresis (SDS-PAGE), and transferred to a Whatman^®^ nitrocellulose membrane (Amersham Protran, GE Healthcare Life Sciences, Enzymatic, Portugal). Membranes were sectioned to allow the detection of multiple protein targets of distinct molecular weights, blocked with 5% skimmed milk or 5% bovine serum albumin (BSA, for γH2AX detection) and probed with specific primary and HRP-conjugated secondary antibodies (described in Supplementary Table S3). Glyceraldehyde 3-phosphate dehydrogenase (GAPDH) or tubulin were used as loading controls. Signal was detected with ECL Amersham (GE Healthcare Life Sciences, Enzymatic, Portugal) using the ChemiDoc^TM^ MP Imaging system (RRID:SCR_019037, Bio Rad Laboratories, Amadora, Portugal). Whole blot images are provided in Supplementary Materials (Figure S3).

### 2.7. Wound Healing Assay

MDA-MB-231 (1 × 10^5^ cells/well), IGROV-1 (6 × 10^4^ cells/well) and PANC-1 (6 × 10^4^ cells/well) were grown to confluence in 2-well silicone culture inserts (Ibidi), and a fixed-width wound was created in the cell monolayer by removing the insert. Cells were treated with DMSO or XGAc in serum-free media and images of the wound were captured at different time points (0, 6, 12, 24, 30 and 48 h) using an inverted NIKON TE 2000-U microscope from Nikon Instruments Inc. (Izasa, Carnaxide, Portugal) at 100× magnification with a DXM1200F digital camera (Nikon Instruments Inc.) and an NIS-Elements microscope imaging software (version 4; Nikon Instruments Inc.). Wound closure was calculated by subtracting the wound area (measured using Fiji Software) at the indicated time points of treatment to the wound area at the starting point.

### 2.8. Combination Therapy

Cells were treated with DMSO (control), the concentration of XGAc that causes 10% growth inhibition (IC_10_, with no significant effect on cell growth) and/or increasing concentrations of olaparib for 48 h. The effect of combined treatments on cell proliferation was analyzed by SRB assay. Mutually nonexclusive combination index (C.I.) and dose reduction index (D.R.I.) were determined using CompuSyn software (RRID:SCR_022931, version 1.0, ComboSyn, Inc., Paramus, NJ, USA), as described [21].

### 2.9. Statistical Analysis

The data presented as mean ± SEM values of at least three independent experiments were analyzed statistically using the GraphPad Prism (La Jolla, CA, USA; version 7.0) software. For comparison of multiple groups, statistical analysis relative to controls was performed using one-way or two-way ANOVA followed by post hoc Sidak’s or Dunnett’s multiple comparison tests. Statistical significance was set as * *p* < 0.05, ** *p* < 0.01, *** *p* < 0.001 and **** *p* < 0.0001.

## 3. Results and Discussion

### 3.1. XGAc Has a Potent Antiproliferative Effect on TNBC, Ovarian Cancer and PDAC Cells, Including Drug-Resistant Cancer Cells

The growth inhibitory activity of XGAc was determined by sulforhodamine B (SRB) assay in a panel of TNBC, ovarian cancer and PDAC cells and compared with olaparib, cisplatin (platinum agent mainly used in breast and ovarian cancer therapy) and gemcitabine (DNA-damaging agent used in pancreatic cancer therapy). The antiproliferative activity of the compounds was also assessed in non-tumorigenic foreskin fibroblasts (HFF-1) and breast (MCF12A) cells to check their selectivity to cancer cells.

In accordance with the half-maximal inhibitory concentration (IC_50_) values, XGAc showed a potent growth inhibitory effect, ranging from 0.25 to 11.13 µM, in TNBC, ovarian cancer and PDAC cells, harbouring either wild-type (wt) or mutBRCA (Figure 1B–D; Supplementary Materials Table S1). Interestingly, regarding PDAC cells, a pronounced antiproliferative activity of XGAc was found in wtBRCA-expressing PDAC cells (IC_50_ values of 0.25 to 0.87 µM), particularly when compared to mutBRCA2-expressing Capan-1 cells (IC_50_ value of 11.13 ± 1.04 µM, Figure 1D).

The effectiveness of XGAc against TNBC, ovarian and PDAC was further reinforced when compared to cisplatin, gemcitabine and olaparib (Figure 1B–D). In particular, XGAc was shown to be much more effective than olaparib regardless of the BRCA status. In fact, olaparib only exhibited a similar antiproliferative effect to XGAc in mutBRCA2 Capan-1 cells (Figure 1D). Importantly, conversely to the standard-of-care agents, XGAc showed selectivity to cancer cells, as demonstrated by its higher IC_50_ values in the non-tumorigenic cells MCF12A (11.33 ± 1.95 µM; Figure 1B) and HFF-1 (30.30 ± 4.19 µM; Figure 1D).

The antiproliferative effect of XGAc in TNBC, ovarian cancer and PDAC cells was further evaluated by colony formation assay (Figure 1E,F). Consistently, also in this assay, a pronounced growth inhibitory effect was observed for XGAc (IC_50_ values of 2.39 ± 0.31 µM in MDA-MB-231, 3.34 ± 0.38 µM in IGROV-1 and 0.20 ± 0.03 µM in PANC-1).

**Figure 2:**
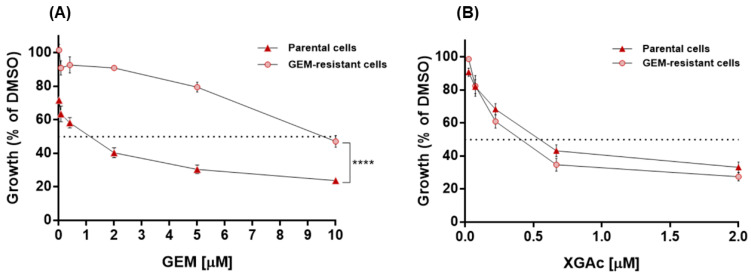
Concentration-response curves for gemcitabine (GEM; (**A**)) and XGAc (**B**) in non-resistant (parental) and GEM-resistant MIA-PaCa-2 cells, after 48 h of treatment by SRB assay. Growth obtained with control (DMSO) was set as 100%. Dotted lines represent the 50% growth inhibition. Data shown are mean ± SEM of three independent experiments (two replicates each); values of GEM-resistant cell growth significantly different from parental cells: **** *p* < 0.0001 (two-way ANOVA followed by Sidak’s test).

XGAc was also tested in gemcitabine (GEM)-resistant MIA-PaCa-2 cells, which showed no cross-resistance to XGAc. In fact, conversely to GEM (Figure 2A), the antiproliferative effect of XGAc in GEM-resistant MIA-PaCa-2 cells (IC_50_ value of 0.40 ± 0.05 µM) was similar to that obtained in non-resistant parental MIA-PaCa-2 cells (IC_50_ value of 0.60 ± 0.07 µM, Figure 2B). These results evidence the effectiveness of XGAc against drug-resistant cancer cells.

**Figure 3:**
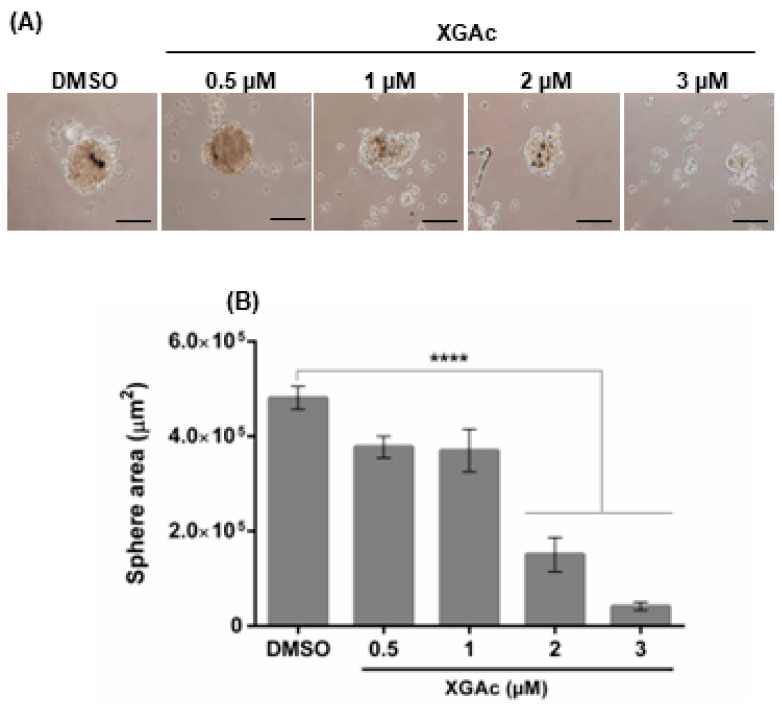
Growth inhibitory effect of XGAc on a 3D model of TNBC cells. Evaluation of HCC1937 mammosphere formation after 72 h of treatment with XGAc; treatment was performed at the seeding time. In (**A**), representative images are shown (scale bar = 50 µm, 100× magnification). In (**B**), quantification of mammosphere area at the end of treatment; data are mean ± SEM of three independent experiments; values significantly different from DMSO: **** *p* < 0.0001 (one-way ANOVA followed by Dunnett’s test).

We further analyzed the effect of XGAc on the formation of a three-dimensional (3D) mammosphere model of the TNBC HCC1937 cells. As observed in Figure 3, after 72 h of treatment with 2 and 3 µM of XGAc (added upon cell seeding), XGAc significantly inhibited spheroid formation (IC_50_ value of 1.42 ± 0.22 µM). Altogether, these results indicated that XGAc inhibits the proliferation and clonogenic potential of cancer cells in 2D and 3D cancer cell models.

### 3.2. XGAc Induces Cell Cycle Arrest, Apoptosis and Genotoxicity, Inhibiting Crucial Players of Homologous Recombination DNA Repair Pathway in TNBC, Ovarian Cancer and PDAC Cells

**Figure 4:**
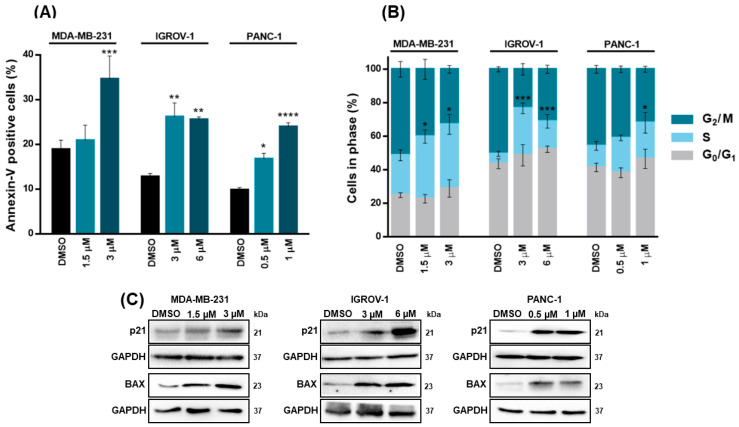
XGAc induces cell cycle arrest and apoptosis in TNBC, ovarian cancer and PDAC cells. Effect of 0.5, 1, 1.5, 3 and 6 µM of XGAc, after 48 h of treatment, on apoptosis (**A**), and cell cycle progression (**B**), in TNBC, ovarian cancer and PDAC cells; data are mean ± SEM of three independent experiments; values significantly different from DMSO: * *p* < 0.05, ** *p* < 0.01, *** *p* < 0.001, **** *p* < 0.0001 (two-way ANOVA followed by Dunnett’s test). (**C**) Effect of XGAc on protein expression levels of p21 and BAX after 48 h treatment. Representative immunoblots are shown; GAPDH was used as a loading control. The uncropped blots are shown in Figure S3.

We next verified that the growth inhibitory effect of XGAc went along with a significant induction of apoptosis (annexin V-positive cells) in MDA-MB-231 (at 3 µM), IGROV-1 (at 3 and 6 µM) and PANC-1 (at 0.5 and 1 µM) cells after 48 h of treatment (Figure 4A). Additionally, after 48 h of treatment, XGAc significantly induced cell cycle arrest at S-phase in MDA-MB-231 (at 1.5 and 3 µM), IGROV-1 (at 3 and 6 µM) and PANC-1 (at 1 µM) (Figure 4B). Consistently, 48 h treatment with XGAc increased the protein expression levels of cell cycle and proapoptotic regulators, p21 and BAX, respectively, in MDA-MB-231, IGROV-1 and PANC-1 (Figure 4C).

In fact, several DNA-targeting agents, such as gemcitabine and irinotecan (a topoisomerase I inhibitor clinically effective against distinct cancers), have shown S-phase-specific cytotoxicity [22,23]. By promoting S-phase arrest, these compounds impair DNA replication [24]. Consistently, previous evidence has indicated that xanthone derivatives may interact with DNA through intercalation, suppressing its replication in cancer cells [11,12].

**Figure 5:**
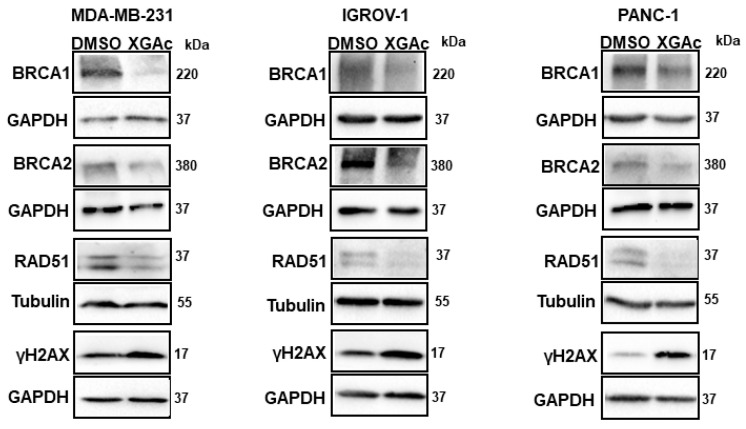
XGAc regulates the expression levels of proteins involved in homologous recombination DNA repair in TNBC, ovarian cancer and PDAC cells. Effect of 1 µM (in PANC-1), 3 µM (in MDA-MB-231) or 6 µM (in IGROV-1) of XGAc on protein expression levels of BRCA1, BRCA2, RAD51 and γH2AX, after 48 h of treatment. Representative immunoblots are shown; GAPDH or tubulin were used as loading controls. The uncropped blots are shown in Figure S3.

Since XGAc suppresses DNA replication in cancer cells by inducing cell cycle arrest in the S-phase, the genotoxic potential of XGAc was evaluated. Accordingly, after 48 h of treatment, XGAc increased the protein expression levels of the phosphorylated histone γH2AX (Figure 5), which is a sensitive marker of DSBs. Altogether, these results indicated that XGAc promoted replication-associated DSBs, stimulating cancer cell death due to unrepaired DNA damage. In conformity with this, it was verified that XGAc could interfere with the expression levels of key proteins in homologous recombination DNA repair, a high-fidelity DNA repair pathway that predominates in the S- and G2-phases of the cell cycle to repair DNA gaps, DSBs and DNA interstrand crosslinks, also providing critical support for DNA replication [25]. In fact, XGAc decreased the protein expression levels of BRCA1, BRCA2 and RAD51 in TNBC (MDA-MB-231 cells; at 3 µM), ovarian cancer (IGROV-1 cells; at 6 µM) and PDAC (PANC-1 cells; at 1 µM) cells after 48 h of treatment (Figure 5).

Particularly, the downregulation of RAD51 by XGAc is highly relevant, since RAD51 is commonly overexpressed in several human malignancies, which is correlated with poor patient survival [26]. In fact, as observed with XGAc, RAD51 inhibitors have been described for their ability to impair human cancer cell growth, induce cell cycle arrest in S-phase and increase the expression levels of γH2AX, also sensitizing cancer cells to other DNA-damaging agents [27].

### 3.3. XGAc Inhibits the Migration of TNBC, Ovarian Cancer and PDAC Cells

**Figure 6:**
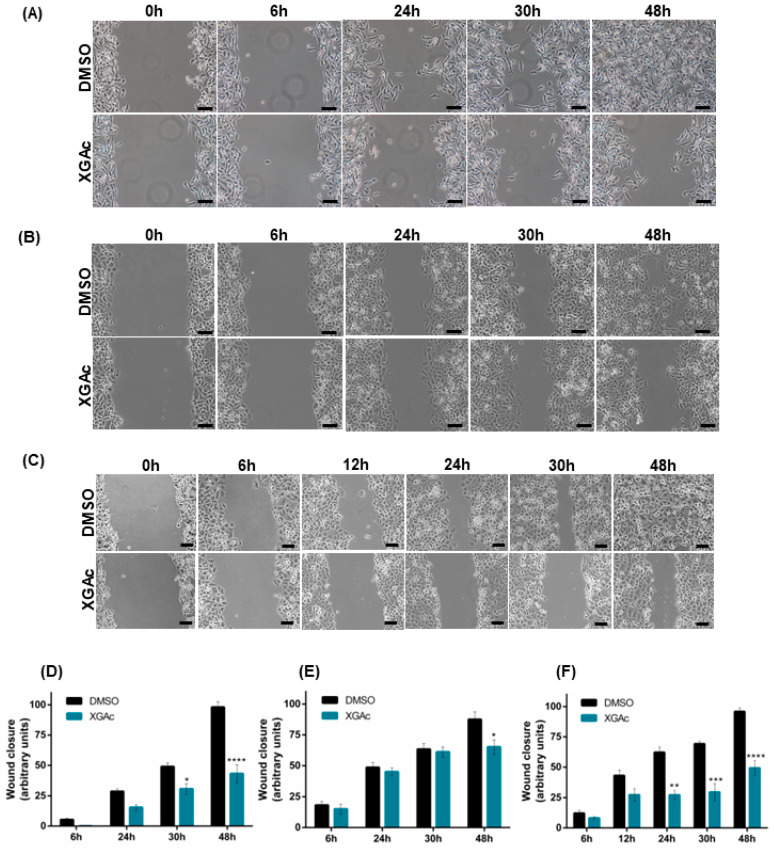
XGAc inhibits TNBC, ovarian cancer and PDAC cell migration. Effect of 47 nM, 200 nM and 16 nM of XGAc (IC_10_ concentration of XGAc in MDA-MB-231, IGROV-1 and PANC-1, respectively) or DMSO on confluent cancer cell migration after 6 h, 12 h, 24 h, 30 h and 48 h of treatment, by a wound healing assay. In (**A**–**C**), representative images are shown (scale bar = 100 µm, 100× magnification) for MDA-MB-231 (**A**), IGROV-1 (**B**) and PANC-1 (**C**). In (**D**–**F**), the quantification of the wound closure is represented for MDA-MB-231 (**D**), IGROV-1 (**E**) and PANC-1 (**F**). Quantification was performed considering randomly selected microscopic fields (four fields per sample), setting the initial wound area as 100%; data are mean ± SEM of three independent experiments; values significantly different from DMSO: * *p* < 0.05, ** *p* < 0.01, *** *p* < 0.001, **** *p* < 0.0001 (two-way ANOVA followed by Sidak’s test).

To evaluate the antimigratory activity of XGAc in TNBC, ovarian cancer and PDAC cells, a wound healing assay was performed. It was observed that 47 nM, 200 nM and 16 nM of XGAc (corresponding to the IC_10_ concentration of XGAc in MDA-MB-231, IGROV-1 and PANC-1, respectively) significantly inhibited cancer cell migration after 24 h (in PANC-1), 30 h (in MDA-MB-231 and PANC-1) and 48 h (in MDA-MB-231, IGROV-1 and PANC-1) of treatment (Figure 6). These results suggested that XGAc suppresses cancer cell motility, potentially preventing tumor dissemination.

Interestingly, in conformity with our work, it was previously reported that the naturally occurring xanthone C-glycoside mangiferin also inhibited the proliferation, migration, and invasion of distinct cancers [28].

### 3.4. XGAc Sensitizes TNBC, Ovarian Cancer and PDAC Cells to Olaparib

**Figure 7:**
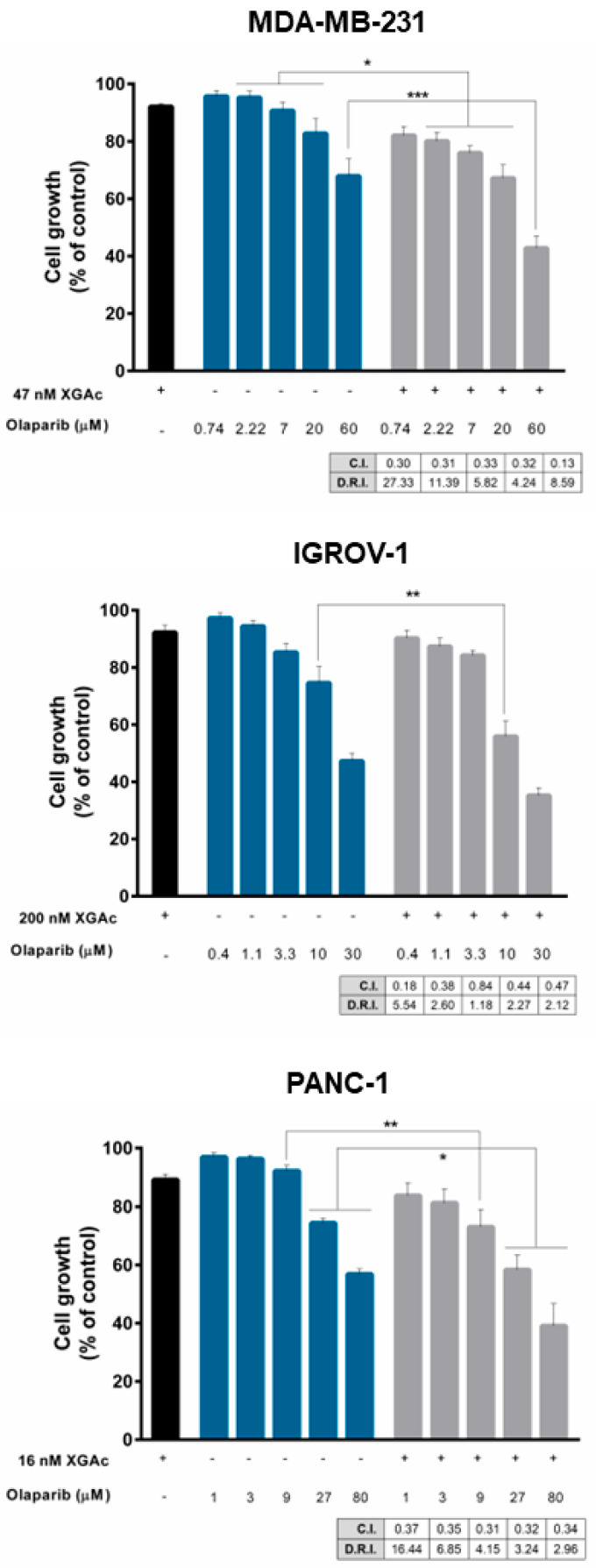
XGAc displays synergistic effects with olaparib in TNBC, ovarian cancer and PDAC cells. Effect of XGAc alone (IC_10_ concentration, represented by a black bar), olaparib alone (represented by blue bars) and XGAc in combination with a concentration range of olaparib (represented by gray bars), on proliferation of cancer cells, determined by SRB assay after 48 h of treatment. Growth obtained with DMSO was set as 100%; data are mean ± SEM of four independent experiments (two replicates each); growth significantly different from olaparib alone: * *p* < 0.05, ** *p* < 0.01, *** *p* < 0.001 (two-way ANOVA followed by Sidak’s test). C.I. and D.R.I. values were obtained using CompuSyn software (C.I. < 1, synergy; 1 < C.I. < 1.1, additive effect; C.I. > 1.1, antagonism).

Since XGAc negatively impacted DNA repair including homologous recombination regulators in cancer cells, we investigated the potential synergistic combination of XGAc with another DNA repair-inhibiting agent, olaparib, that compromises DNA single-strand break repair, affecting DNA repair pathways such as base excision repair and non-homologous end joining. For that, a single concentration of XGAc, with no significant effect on cancer cell growth (IC_10_), and a concentration range of olaparib were tested in cancer cells. The results revealed that XGAc enhanced the growth inhibitory activity of olaparib when compared to olaparib alone, either in wt or mutBRCA cancer cells (Figure 7). The combination index (C.I.) and dose reduction index (D.R.I.) values were determined by multiple drug-effect analyses for each combination, showing synergistic effects between XGAc and olaparib for all the combinations tested (C.I. < 1), and a marked reduction in the effective dose of olaparib (Figure 7). Particularly, in MDA-MB-231, the synergistic combination of 47 nM XGAc and 0.74 µM olaparib (C.I. of 0.30) was associated with 27.33-fold reduction of the effective dose of olaparib; in IGROV-1, a 5.54-fold reduction was achieved with 200 nM XGAc and 0.4 µM olaparib (C.I. of 0.18); and in PANC-1, the 16 nM XGAc and 1 µM olaparib synergistic combination (C.I. of 0.37) caused a 16.44-fold reduction of the effective dose of olaparib (Figure 7).

The clinical use of PARPis in combination with DNA-damaging agents is limited by the more-than-addictive cytotoxicity [29]. However, XGAc exhibited synergistic effects with olaparib, which may be explained by a synthetic lethality effect involving an inhibition of homologous recombination DNA repair by XGAc and of single-strand break repair by olaparib. In fact, the mutual inhibition of different DNA repair pathways significantly compromises cell survival [30]. Consistently, previous works have shown synergistic effects between olaparib and novel homologous recombination DNA repair inhibitors capable of inducing a BRCAness phenotype, namely, in PDAC cells [31,32,33].

The synergism with XGAc will reduce the effective dose of olaparib and, subsequently, its undesirable toxicity. Also, since resistance to olaparib is a major clinical concern, the combination with XGAc will (re)sensitize cancer cells to its cytotoxic effect. Importantly, this synergistic combination may also be applied to homologous recombination-proficient patients since XGAc enhanced the growth inhibitory activity of olaparib in wtBRCA cancer cells, which will extend the population of cancer patients that may respond to this targeted anticancer drug. In summary, these findings indicate that XGAc may enhance the sensitivity of cancer cells to olaparib by potentially inhibiting homologous recombination.

Despite its clinical approval, the limitations of olaparib are known, particularly due to its inability to trigger synthetic lethality in homologous recombination-proficient patients. The ability of XGAc to induce synthetic lethality when combined with olaparib, increasing its efficacy, and extending the population of patients that may benefit from this drug, has high clinical relevance and is worthy of this study. Although it has not been studied in this work, the mechanism of action of this compound leads us to also predict promising synergistic effects of XGAc with other chemotherapeutic agents, such as cisplatin and gemcitabine.

### 3.5. XGAc Induces Cytotoxicity in Patient-Derived Ovarian Cancer Cells

**Figure 8:**
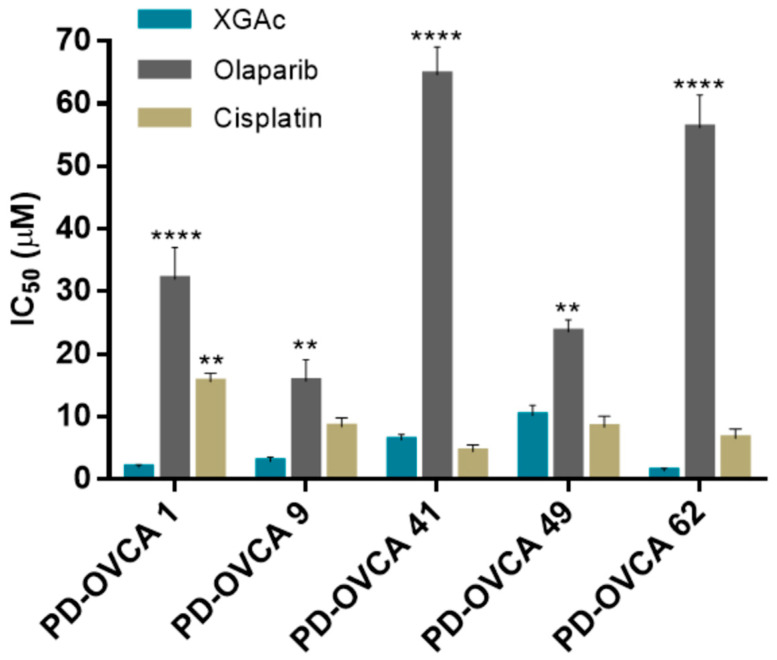
XGAc has a potent cytotoxic effect in patient-derived ovarian cancer cells (PD-OVCA). IC_50_ values of XGAc, olaparib and cisplatin in PD-OVCA cancer cells with pathogenic mut*BRCA1* (PD-OVCA 1 and 9), benign mut*BRCA1* (PD-OVCA 41) or wt*BRCA1* (PD-OVCA 49 and 62), determined by MTS assay after 48 h of treatment. Data are mean ± SEM of five independent experiments; values significantly different from XGAc: ** *p* < 0.01, **** *p* < 0.0001 (two-way ANOVA followed by Dunnett’s test).

The cytotoxic effect of XGAc was further evaluated in patient-derived ovarian cancer cells harbouring wt or mut*BRCA1* (Supplementary Materials, Table S2) by MTS assay. Consistently with our previous results (Figure 1), regardless of *BRCA1* status, XGAc was much more effective than olaparib in reducing the cell viability of all patient-derived ovarian cancer cells (Figure 8). Notably, XGAc was also more effective than cisplatin in platinum-resistant patient-derived PD-OVCA 1 cells harbouring a pathogenic mut*BRCA1* (Figure 8; Supplementary Materials, Table S2).

As observed in GEM-resistant MIA-PaCa-2 cells (Figure 2), the promising cytotoxicity obtained with XGAc in PD-OVCA 1 (post-chemo platinum-resistant patient-derived ovarian cancer cells) indicated that these cells showed no cross-resistance to XGAc (Figure 8). PD-OVCA 1 was derived from a patient that received different treatments based on carboplatin, followed by gemcitabine and topotecan, and finally mitoxantrone, developing post-chemo platinum resistance [17]. In fact, drug effectiveness is often limited by the emergence of cancer cell resistance [34]. However, these results corroborated the potential of XGAc against drug-resistant cancer cells.

Altogether, these results confirmed the efficacy of XGAc against both wt and mutBRCA1 cancer cell lines. In fact, the higher cytotoxic effect of XGAc could be observed in mut*BRCA1* PD-OVCA 1 and wt*BRCA1* PD-OVCA 62 cells (Figure 8).

It is worth noting that the low sensitivity of PD-OVCA 41 to olaparib may be related to the presence, in this patient-derived cell line, of a benign missense mut*BRCA1*. On the other hand, the notable sensitivity of wt*BRCA1* PD-OVCA 49 to olaparib suggests some homologous recombination impairment in this cell line, namely, in non-*BRCA* genes (BRCAness phenotype); however, we do not have further information to confirm this hypothesis.

## 4. Conclusions

This work reports the promising antitumor activity of XGAc in TNBC, ovarian and PDAC cancer cells. Herein, we demonstrated that XGAc decreased cancer cell proliferation, viability, and motility, exhibiting low cytotoxicity against normal cells, and being much more effective than olaparib regardless of BRCA status. Importantly, XGAc was shown to be highly effective in drug-resistant cancer cells, suggesting its great therapeutic relevance in resistant and hard-to-treat cancers.

PARPis are currently on the frontline of targeted therapy for mutBRCA patients. However, its efficacy in monotherapy is limited to mutBRCA cancer patients and cancer cells frequently become resistant to this therapy by restoring or retaining a residual DNA repair machinery. Thus, the development of strategies that improve the therapeutic potential of PARPis and allow the overcoming of resistance to these agents, namely, through combination therapy is of great relevance. Following this premise, the inhibition of homologous recombination DNA repair constitutes a promising anticancer strategy particularly to (re)sensitize cancer cells to DNA-damaging agents. Herein, XGAc was identified as a genotoxic xanthone derivative, promoting replication-associated DSBs in cancer cells and triggering apoptotic cancer cell death by DNA repair inhibition. XGAc may induce synthetic lethality effects with PARPis that negatively impact single-strand break repair. In fact, XGAc sensitized both wt and mutBRCA cancer cells to the effect of olaparib, extending the population of cancer patients that may respond to this targeted therapy while reducing its effective dose and subsequent toxic side effects.

In conclusion, this work supports the promising application of XGAc in the treatment of hard-to-treat cancers, either alone or in combination with olaparib. Importantly, these results pave the way to future works that may explore acetylated xanthonosides as anticancer agents, particularly by their DNA-targeting ability. Moreover, it encourages combination therapy to induce synthetic lethality in cancer cells, sensitizing them to the standard-of-care therapy.

## Data Availability

The data can be shared up on request.

## Supplementary Materials

The following supporting information can be downloaded at: https://www.mdpi.com/article/10.3390/cancers15245718/s1, Table S1: Characterization of human immortalized normal and cancer cells; Figure S1: Concentration-response curves for the growth inhibitory effect of XGAc on TNBC (A), ovarian cancer (B) and PDAC cells (C), including the normal cells MCF12A (A) and HFF-1 (C); Table S2: Characterization of patient-derived ovarian cancer (PD-OVCA) cells; Figure S2: Concentration-response curves for the growth inhibitory effect of XGAc on patient-derived ovarian cancer (PD-OVCA) cells; Table S3: List of antibodies used in Western blot; Figure S3: Whole blot images.

## References

[1] Mekonnen N., Yang H., Shin Y.K. (2022). Homologous Recombination Deficiency in Ovarian, Breast, Colorectal, Pancreatic, Non-Small Cell Lung and Prostate Cancers, and the Mechanisms of Resistance to PARP Inhibitors. Front. Oncol..

[2] Raimundo L., Calheiros J., Saraiva L. (2021). Exploiting DNA Damage Repair in Precision Cancer Therapy: BRCA1 as a Prime Therapeutic Target. Cancers.

[3] Calheiros J., Corbo V., Saraiva L. (2023). Overcoming Therapeutic Resistance in Pancreatic Cancer: Emerging Opportunities by Targeting BRCAs and P53. Biochim. Biophys. Acta Rev. Cancer.

[4] Abbotts R., Dellomo A.J., Rassool F.V. (2022). Pharmacologic Induction of BRCAness in BRCA-Proficient Cancers: Expanding PARP Inhibitor Use. Cancers.

[5] Luo L., Keyomarsi K. (2022). PARP Inhibitors as Single Agents and in Combination Therapy: The Most Promising Treatment Strategies in Clinical Trials for BRCA-Mutant Ovarian and Triple-Negative Breast Cancers. Expert Opin. Investig. Drugs.

[6] Jonsson P., Bandlamudi C., Cheng M.L., Srinivasan P., Chavan S.S., Friedman N.D., Rosen E.Y., Richards A.L., Bouvier N., Selcuklu S.D. (2019). Tumour Lineage Shapes BRCA-Mediated Phenotypes. Nature.

[7] Ragupathi A., Singh M., Perez A.M., Zhang D. (2023). Targeting the BRCA1/2 Deficient Cancer with PARP Inhibitors: Clinical Outcomes and Mechanistic Insights. Front. Cell Dev. Biol..

[8] Liu Y., Burness M.L., Martin-Trevino R., Guy J., Bai S., Harouaka R., Brooks M.D., Shang L., Fox A., Luther T.K. (2017). RAD51 Mediates Resistance of Cancer Stem Cells to PARP Inhibition in Triple-Negative Breast Cancer. Clin. Cancer Res..

[9] Cruz C., Castroviejo-Bermejo M., Gutiérrez-Enríquez S., Llop-Guevara A., Ibrahim Y.H., Gris-Oliver A., Bonache S., Morancho B., Bruna A., Rueda O.M. (2018). RAD51 Foci as a Functional Biomarker of Homologous Recombination Repair and PARP Inhibitor Resistance in Germline BRCA-Mutated Breast Cancer. Ann. Oncol..

[10] Gomes A.S., Brandão P., Fernandes C.S.G., da Silva M.R.P.C., de da Sousa M.E.S.P., de Pinto M.M.M. (2016). Drug-like Properties and ADME of Xanthone Derivatives: The Antechamber of Clinical Trials. Curr. Med. Chem..

[11] Wang H., Wei L., Yan H., Gao X., Xu B., Tang N. (2013). Antitumor Activity and DNA-Binding Investigations of Isoeuxanthone and Its Piperidinyl Derivative. Chem. Pharm. Bull..

[12] Uvarani C., Arumugasamy K., Chandraprakash K., Sankaran M., Ata A., Mohan P.S. (2015). A New DNA-Intercalative Cytotoxic Allylic Xanthone from Swertia Corymbosa. Chem. Biodivers..

[13] Wezeman T., Bräse S., Masters K.-S. (2015). Xanthone Dimers: A Compound Family Which Is Both Common and Privileged. Nat. Prod. Rep..

[14] Suda I., Oki T., Masuda M., Nishiba Y., Furuta S., Matsugano K., Sugita K., Terahara N. (2002). Direct Absorption of Acylated Anthocyanin in Purple-Fleshed Sweet Potato into Rats. J. Agric. Food Chem..

[15] Alves A., Correia-da-Silva M., Nunes C., Campos J., Sousa E., Silva P.M.A., Bousbaa H., Rodrigues F., Ferreira D., Costa P.C. (2019). Discovery of a New Xanthone against Glioma: Synthesis and Development of (Pro)Liposome Formulations. Molecules.

[16] Ragone A., Salzillo A., Spina A., Naviglio S., Sapio L. (2022). Integrating Gemcitabine-Based Therapy With AdipoRon Enhances Growth Inhibition in Human PDAC Cell Lines. Front. Pharmacol..

[17] Indraccolo S., Tisato V., Agata S., Moserle L., Ferrari S., Callegaro M., Persano L., Palma M.D., Scaini M.C., Esposito G. (2006). Establishment and Characterization of Xenografts and Cancer Cell Cultures Derived from BRCA1 −/− Epithelial Ovarian Cancers. Eur. J. Cancer.

[18] Percie du Sert N., Hurst V., Ahluwalia A., Alam S., Avey M.T., Baker M., Browne W.J., Clark A., Cuthill I.C., Dirnagl U. (2020). The ARRIVE Guidelines 2.0: Updated Guidelines for Reporting Animal Research. Br. J. Pharmacol..

[19] Raimundo L., Paterna A., Calheiros J., Ribeiro J., Cardoso D.S.P., Piga I., Neto S.J., Hegan D., Glazer P.M., Indraccolo S. (2021). BBIT20 Inhibits Homologous DNA Repair with Disruption of the BRCA1–BARD1 Interaction in Breast and Ovarian Cancer. Br. J. Pharmacol..

[20] Schindelin J., Arganda-Carreras I., Frise E., Kaynig V., Longair M., Pietzsch T., Preibisch S., Rueden C., Saalfeld S., Schmid B. (2012). Fiji: An Open-Source Platform for Biological-Image Analysis. Nat. Methods.

[21] Chou T.C., Talalay P. (1984). Quantitative Analysis of Dose-Effect Relationships: The Combined Effects of Multiple Drugs or Enzyme Inhibitors. Adv. Enzyme Regul..

[22] Xu Y., Villalona-Calero M.A. (2002). Irinotecan: Mechanisms of Tumor Resistance and Novel Strategies for Modulating Its Activity. Ann. Oncol..

[23] Beutel A.K., Halbrook C.J. (2023). Barriers and Opportunities for Gemcitabine in Pancreatic Cancer Therapy. Am. J. Physiol. Cell Physiol..

[24] Li Y., Yu H., Han F., Wang M., Luo Y., Guo X. (2018). Biochanin A Induces S Phase Arrest and Apoptosis in Lung Cancer Cells. Biomed. Res. Int..

[25] Chakraborty S., Schirmeisen K., Lambert S.A.E. (2023). The Multifaceted Functions of Homologous Recombination in Dealing with Replication-Associated DNA Damages. DNA Repair.

[26] Saeki H., Jogo T., Kawazoe T., Kamori T., Nakaji Y., Zaitsu Y., Fujiwara M., Baba Y., Nakamura T., Iwata N. (2022). RAD51 Expression as a Biomarker to Predict Efficacy of Preoperative Therapy and Survival for Esophageal Squamous Cell Carcinoma: A Large-Cohort Observational Study (KSCC1307). Ann. Surg..

[27] Gu P., Xue L., Zhao C., Li W., Jiang Z., Liu A., Li T., Liu L., Decker M., Cheng X. (2022). Targeting the Homologous Recombination Pathway in Cancer With a Novel Class of RAD51 Inhibitors. Front. Oncol..

[28] Gold-Smith F., Fernandez A., Bishop K. (2016). Mangiferin and Cancer: Mechanisms of Action. Nutrients.

[29] Yap T.A., Plummer R., Azad N.S., Helleday T. (2019). The DNA Damaging Revolution: PARP Inhibitors and Beyond. Am. Soc. Clin. Oncol. Educ. Book.

[30] Bryant H.E., Schultz N., Thomas H.D., Parker K.M., Flower D., Lopez E., Kyle S., Meuth M., Curtin N.J., Helleday T. (2005). Specific Killing of BRCA2-Deficient Tumours with Inhibitors of Poly(ADP-Ribose) Polymerase. Nature.

[31] Falchi F., Giacomini E., Masini T., Boutard N., Di Ianni L., Manerba M., Farabegoli F., Rossini L., Robertson J., Minucci S. (2017). Synthetic Lethality Triggered by Combining Olaparib with BRCA2–Rad51 Disruptors. ACS Chem. Biol..

[32] Roberti M., Schipani F., Bagnolini G., Milano D., Giacomini E., Falchi F., Balboni A., Manerba M., Farabegoli F., De Franco F. (2019). Rad51/BRCA2 Disruptors Inhibit Homologous Recombination and Synergize with Olaparib in Pancreatic Cancer Cells. Eur. J. Med. Chem..

[33] Bagnolini G., Milano D., Manerba M., Schipani F., Ortega J.A., Gioia D., Falchi F., Balboni A., Farabegoli F., De Franco F. (2020). Synthetic Lethality in Pancreatic Cancer: Discovery of a New RAD51-BRCA2 Small Molecule Disruptor That Inhibits Homologous Recombination and Synergizes with Olaparib. J. Med. Chem..

[34] Gupta R.S., Murray W., Gupta R. (1988). Cross Resistance Pattern towards Anticancer Drugs of a Human Carcinoma Multidrug-Resistant Cell Line. Br. J. Cancer.

