# Supplementary material for: Antitumor Activity of the Xanthonoside XGAc in Triple-Negative Breast, Ovarian and Pancreatic Cancer by Inhibiting DNA Repair
Source: Cancers (Basel). 2023 Dec 6;15(24):5718. doi: 10.3390/cancers15245718 (PMC10741784; doi:10.3390/cancers15245718)
Supplement: Supplementary file 1 [file cancers-15-05718-s001.zip › cancers-2676061-supplementary.pdf]

Supplementary material

Antitumor activity of the xanthonoside XGAc in triple-negative breast, ovarian and pancreatic cancer by inhibiting DNA repair

Juliana Calheiros, Liliana Raimundo, João Morais, Ana Catarina Matos, Sonia Anna Minuzzo, Stefano Indraccolo, Emília Sousa, Marta Correia da Silva and Lucília Saraiva

Table S1. Characterization of human immortalized normal and cancer cells.

Cell line	RRID	Tissue/disease	BRCA status	Supplier
**MDA-MB-231**	ATCC Cat# CRL-12532, RRID:CVCL_0062	TNBC	wt with BRCA1 LOH	ATCC (Rockville, MD, USA)
**MDA-MB-468**	ATCC Cat# HTB-132, RRID:CVCL_0419		wt with BRCA1 LOH	
**HCC1937**	ATCC Cat# CRL-2336, RRID:CVCL_0290		Homozygous 5382insC mutBRCA1	
**MCF12A**	ATCC Cat# CRL-3598, RRID:CVCL_3744	Non-tumorigenic breast cells	wt	
**OVCAR-3**	ATCC Cat# HTB-161, RRID:CVCL_0465	HGSOC	wt	
**SKOV-3**	ATCC Cat# HTB-77, RRID:CVCL_0532	Ovarian serous cystadenocarcinoma	wt	
**IGROV-1**	RRID:CVCL_1304	Ovarian endometrioid adenocarcinoma	Heterozygous 280delA mutBRCA1, and p.Lys1108fs mutBRCA2	Provided by Prof Leonor David from Instituto de Investigação e Inovação em Saúde, i3s, Porto, Portugal
**PANC-1**	ATCC Cat# CRL-1469, RRID:CVCL_0480	Pancreatic ductal adenocarcinoma	wt	ATCC (Rockville, MD, USA)
**MIA-PaCa-2 GEM-resistant**	ATCC Cat# CRL-1420, RRID:CVCL_0428		wt	Provided by Professor Luigi Sapio from Università degli Studi

				della Campania, Italy
**MIA-PaCa-2**				
**AsPC1**	ATCC Cat# CRL-1682, RRID:CVCL_0152		wt	ATCC (Rockville, MD, USA)
**BxPC3**	ATCC Cat# CRL-1687, RRID:CVCL_0186		wt	
**Hs766T**	ATCC Cat# HTB-134, RRID:CVCL_0334		wt	
**HPAF-II**	ATCC Cat# CRL-1997, RRID:CVCL_0313		wt	
**Capan-1**	ATCC Cat# HTB-79, RRID:CVCL_0237		Hemizygous 6174delT mutBRCA2	
**HFF-1**	ATCC Cat# SCRC-1041, RRID:CVCL_3285	Non-tumorigenic Foreskin fibroblasts	wt	

HGSOC, high grade papillary serous ovarian carcinoma; LOH, loss of heterozigoty; wt, wild-type

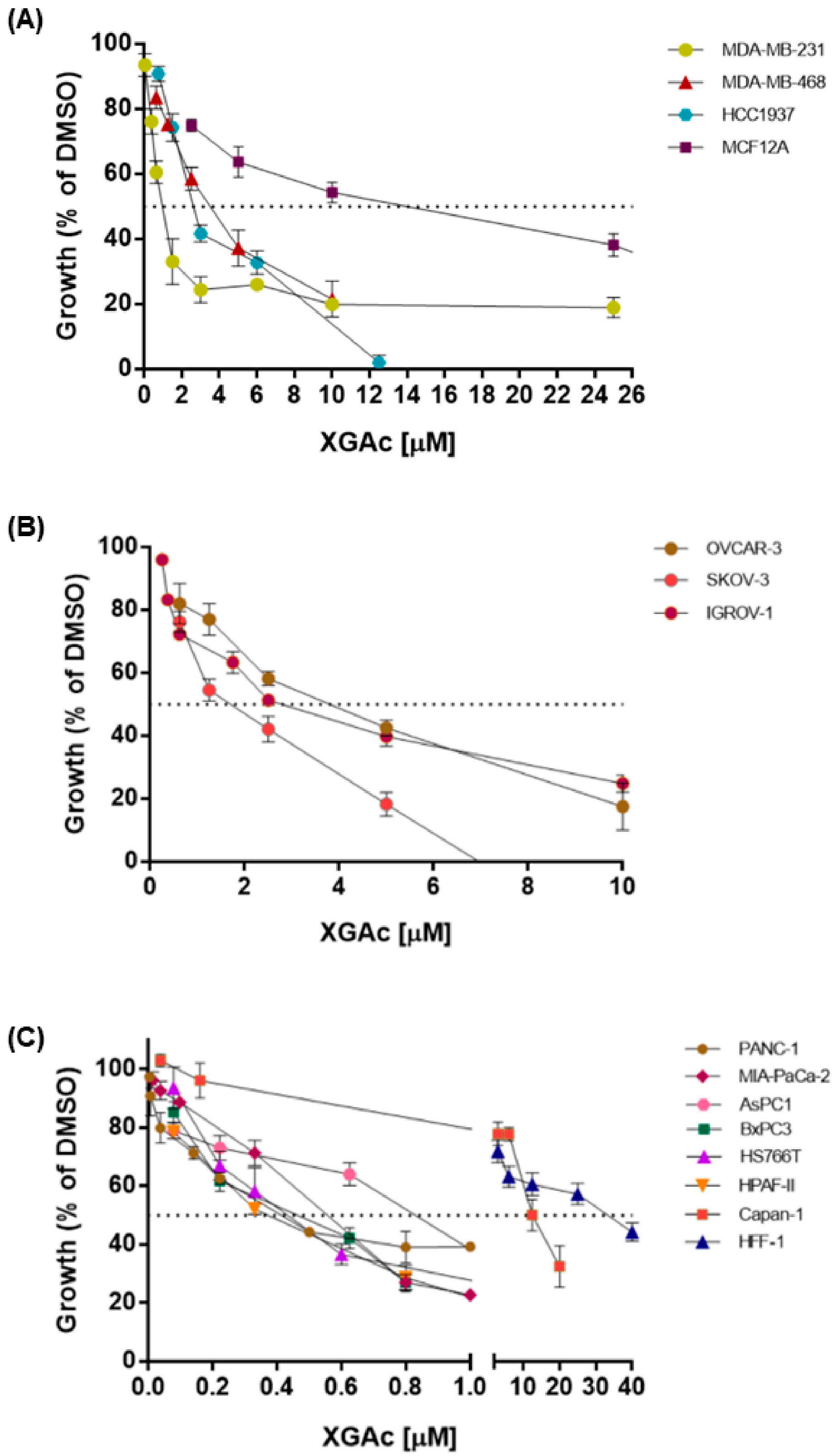

Figure S1. Concentration-response curves for the growth inhibitory effect of XGAc on TNBC (A), ovarian cancer (B) and PDAC cells (C), including the normal cells MCF12A (A) and HFF-1 (C), determined by SRB assay after 48 h treatment; growth obtained with control was set as 100%; data are mean ± SEM, n=3 independent experiments (two replicates each).

Table S2. Characterization of patient-derived ovarian cancer (PD-OVCA) cells.

Cell line	Tissue/disease	BRCA1 status	Therapy
**PD-OVCA 1**	HGSOC stage IV/Grade III	Germline missense pathogenic mutation (lack of exon 17)	Post-chemo: platinum resistant
**PD-OVCA 9**	Ovarian adenocarcinoma	Somatic nonsense pathogenic mutation at exon 13 (c.4389C>G; p.Y1463X)<br>Somatic benign mutation at exon 13 (c.4390C>T; p.P1464S)	Post-chemo: platinum sensitive
**PD-OVCA 41**	Endometrioid carcinoma	Somatic missense benign mutation at exon 10 (c.2077G>A; p.D693N)	Post-chemo
**PD-OVCA 49**	HGSOC stage IV/Grade III	wt	Post-chemo
**PD-OVCA 62**	HGSOC	wt	Post-chemo

HGSOC, high grade papillary serous ovarian carcinoma; wt, wild-type

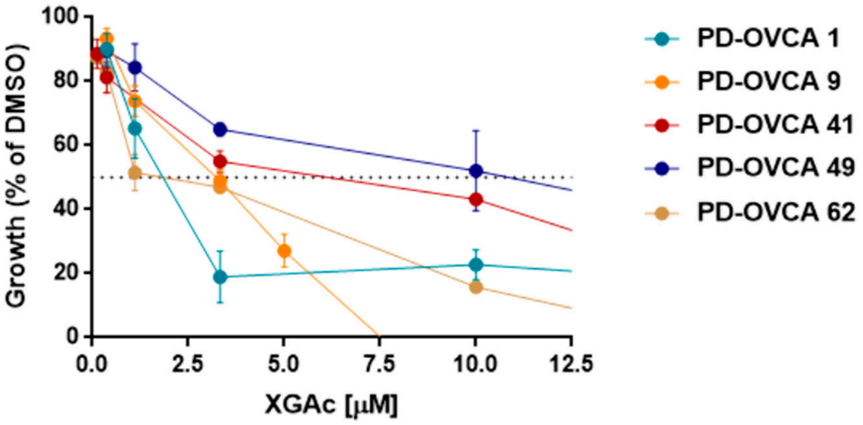

Figure S2. Concentration-response curves for the growth inhibitory effect of XGAc on patient-derived ovarian cancer (PD-OVCA) cells determined after 48 h treatment by MTS assay; growth obtained with control was set as 100%; data are mean ± SEM of five independent experiments.

Table S3. List of antibodies used in western blot.

Antigen	Blocking solution	Dilution	Supplier	Cat#; RRID
**Primary antibodies**				
**GAPDH (6C5)** mouse mAb	5% skimmed milk	1:10000	Santa Cruz Biotechnology	sc-32233; RRID:AB_627679
**α-Tubulin (B-7)** mouse mAb		1:500		sc-5286; RRID:AB_628411
**p21 (F-5)** mouse mAb		1:100		sc-6246; RRID:AB_628073
**BAX (B-9)** mouse mAb		1:200		sc-7480; RRID:AB_626729
**γH2AX (phospho-Ser139)** rabbit mAb	5% bovine serum albumin	1:10000	Abcam	ab2893; RRID:AB_303388
**RAD51 [EPR4030(3)] r**abbit mAb	5% skimmed milk	1:500		ab133534; RRID:AB_2722613
**BRCA1 (A8X9F)** rabbit mAb		1:1000	Cell signaling	14823; RRID:AB_2798631
**BRCA2 (D9S6V)** rabbit mAb		1:800		10741; RRID:AB_2797730
**Secondary antibodies**				
**Anti-mouse** HRP-conjugated	5% skimmed milk	1:5000	Santa Cruz Biotechnology	sc-2005; RRID:AB_631736
**Anti-rabbit** HRP-conjugated		1:5000		sc-2357; RRID:AB_628497

GAPDH, glyceraldehyde 3-phosphate dehydrogenase; HRP, horseradish peroxidase

Figure S3. Whole blot images

Samples for other non-related work:

NR

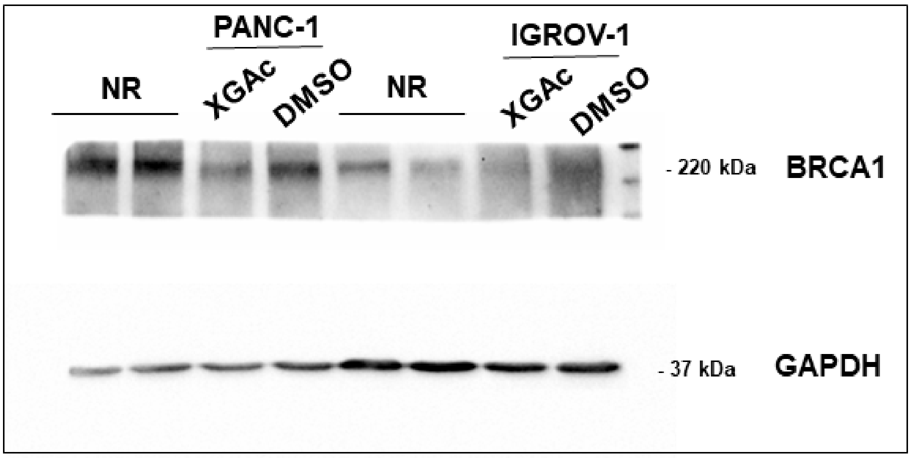

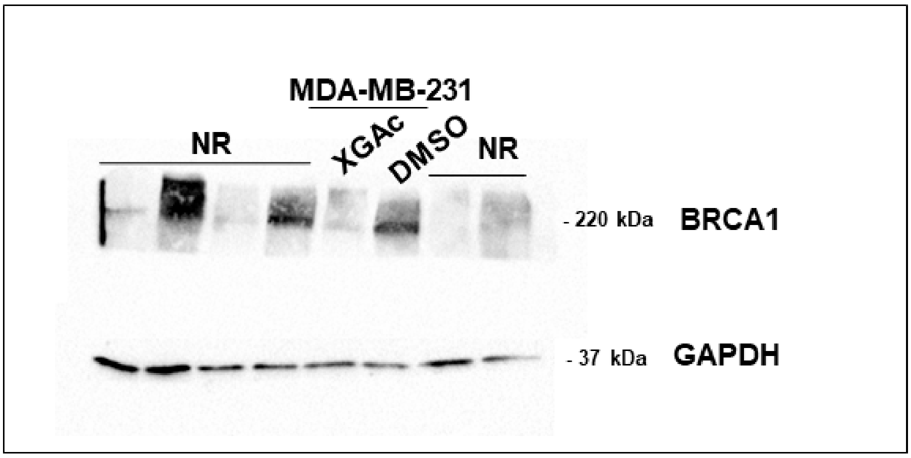

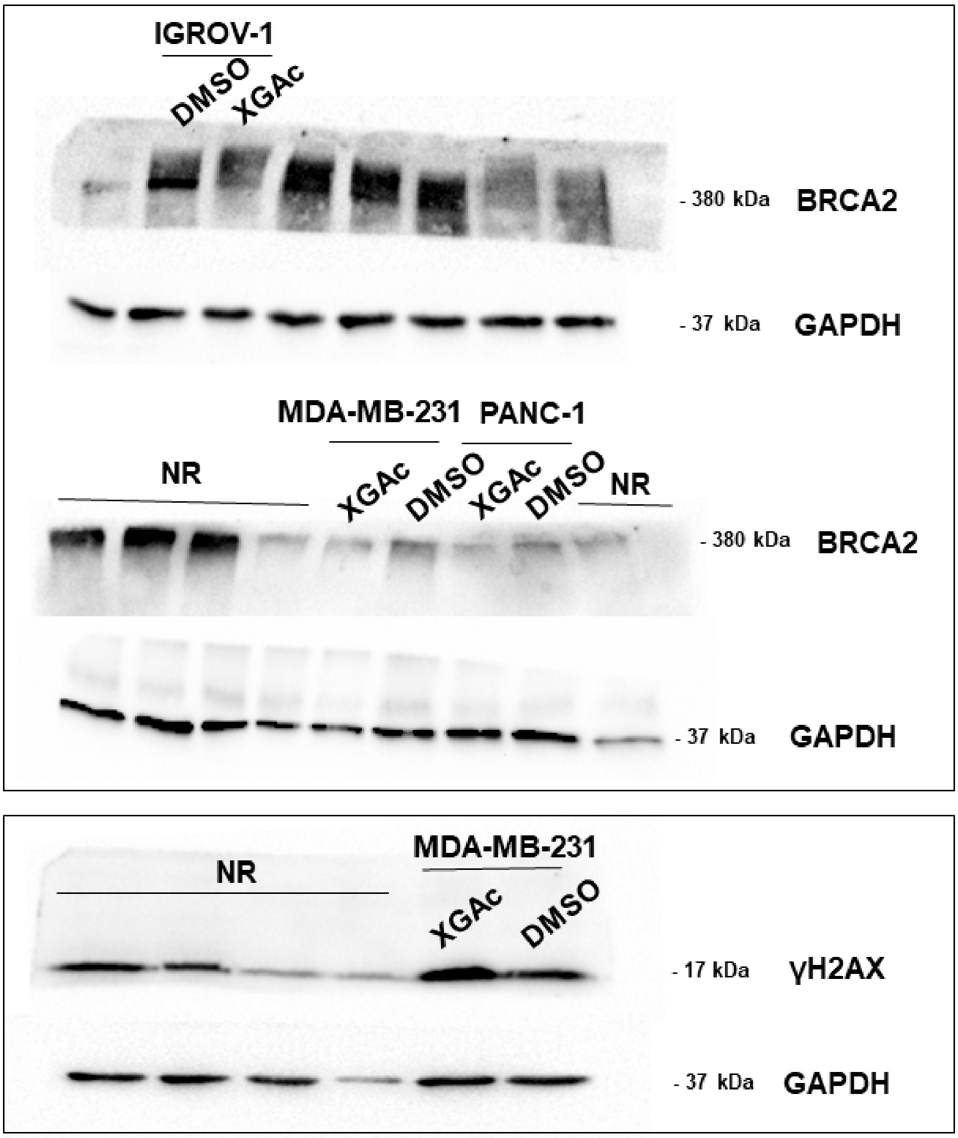
IGROV-1
DMSO
XGAc
- 380 kDa
BRCA2
- 37 kDa
GAPDH
MDA-MB-231
PANC-1
NR
XGAc
DMSO
XGAc
DMSO
NR
- 380 kDa
BRCA2
- 37 kDa
GAPDH
NR
MDA-MB-231
XGAc
DMSO
- 17 kDa
γH2AX
- 37 kDa
GAPDH

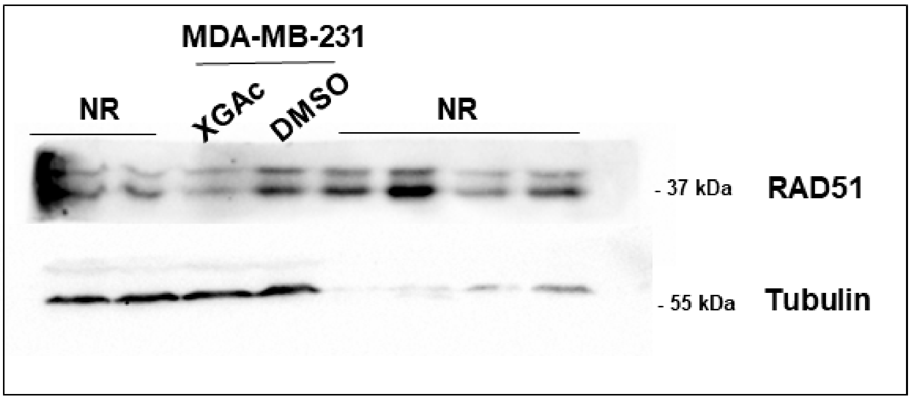
MDA-MB-231
NR
XGAc
DMSO
NR
- 37 kDa
RAD51
- 55 kDa
Tubulin

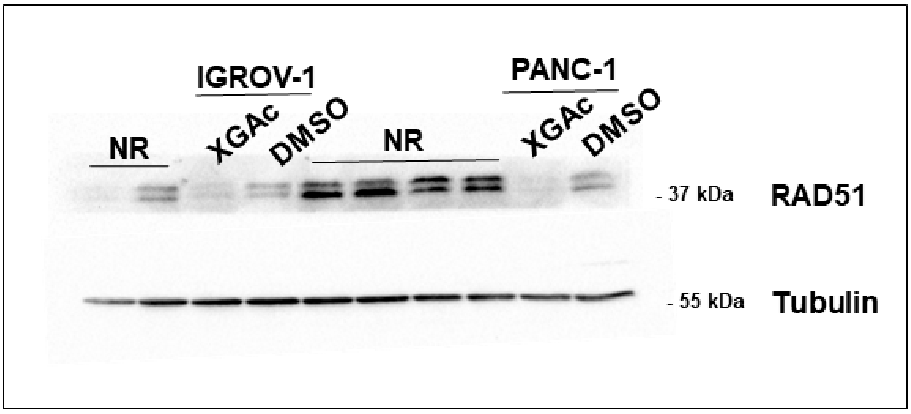
IGROV-1
PANC-1
NR
XGAc
DMSO
NR
XGAc
DMSO
- 37 kDa
RAD51
- 55 kDa
Tubulin

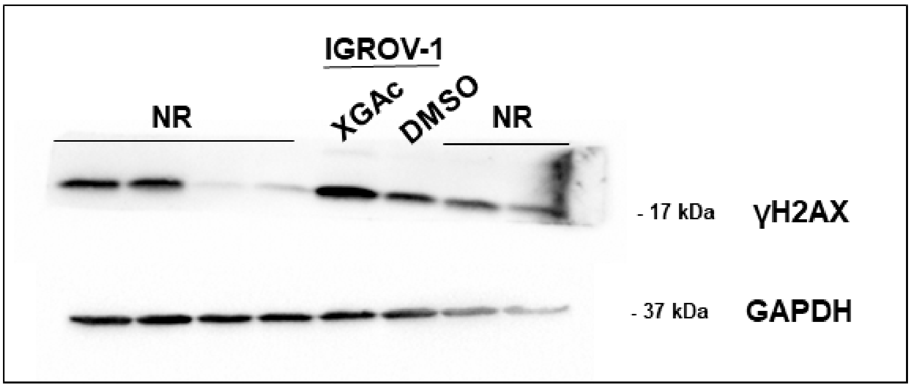
IGROV-1
NR
XGAc
DMSO
NR
- 17 kDa
γH2AX
- 37 kDa
GAPDH

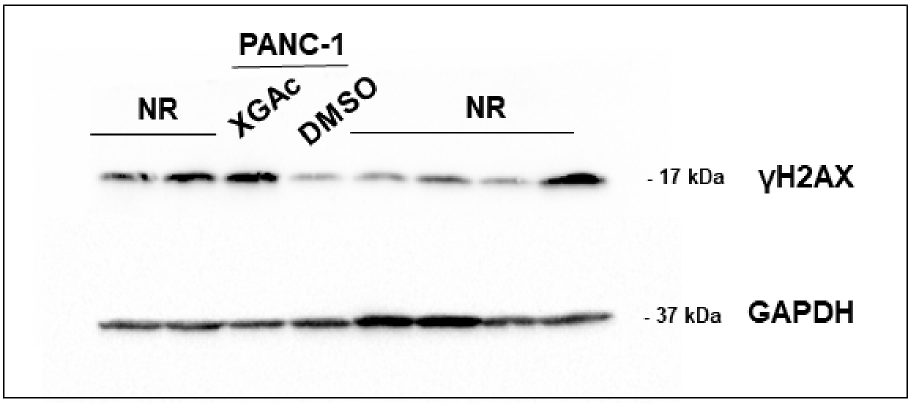
PANC-1
NR
XGAc
DMSO
NR
- 17 kDa
γH2AX
- 37 kDa
GAPDH

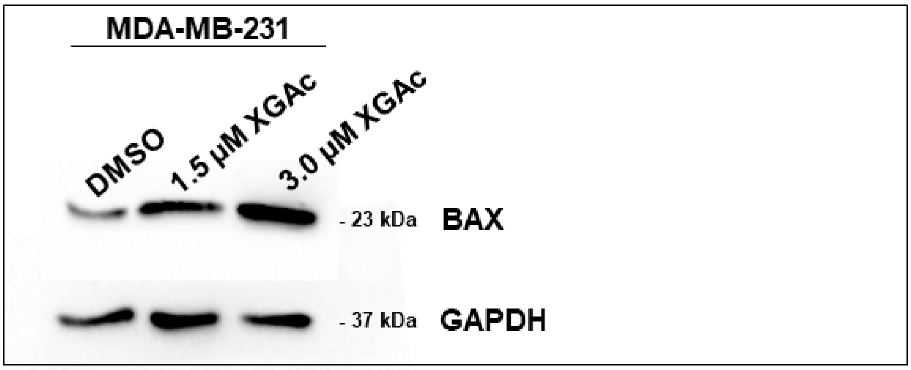
MDA-MB-231
DMSO
1.5 µM XGAc
3.0 µM XGAc
- 23 kDa
BAX
- 37 kDa
GAPDH

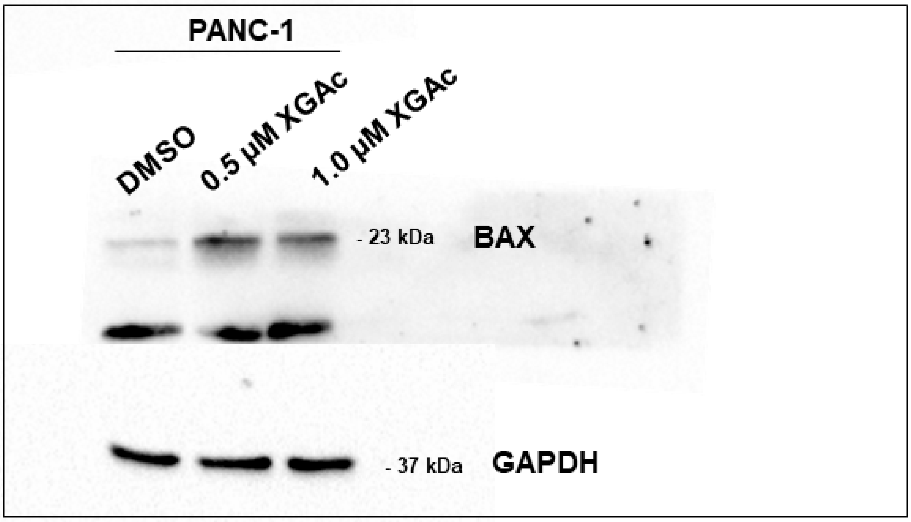
PANC-1
DMSO
0.5 µM XGAc
1.0 µM XGAc
- 23 kDa
BAX
- 37 kDa
GAPDH

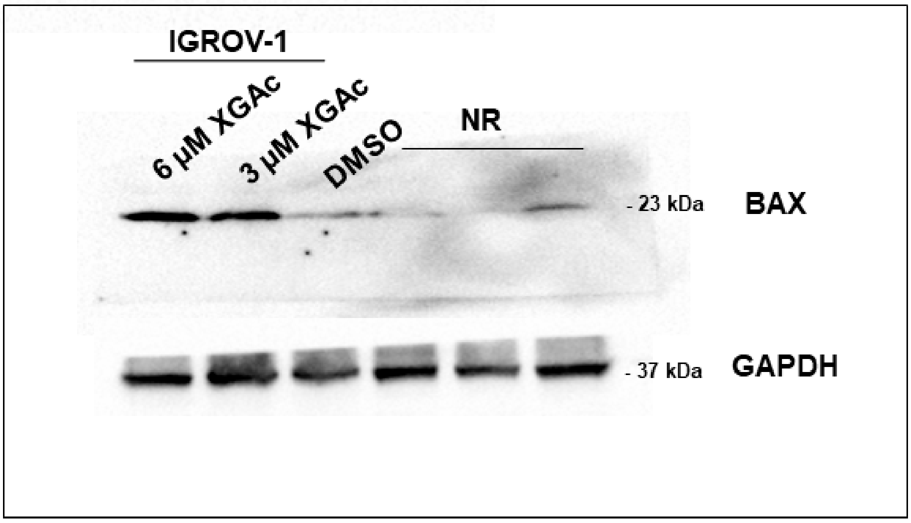
IGROV-1
6 µM XGAc
3 µM XGAc
DMSO
NR
- 23 kDa
BAX
- 37 kDa
GAPDH

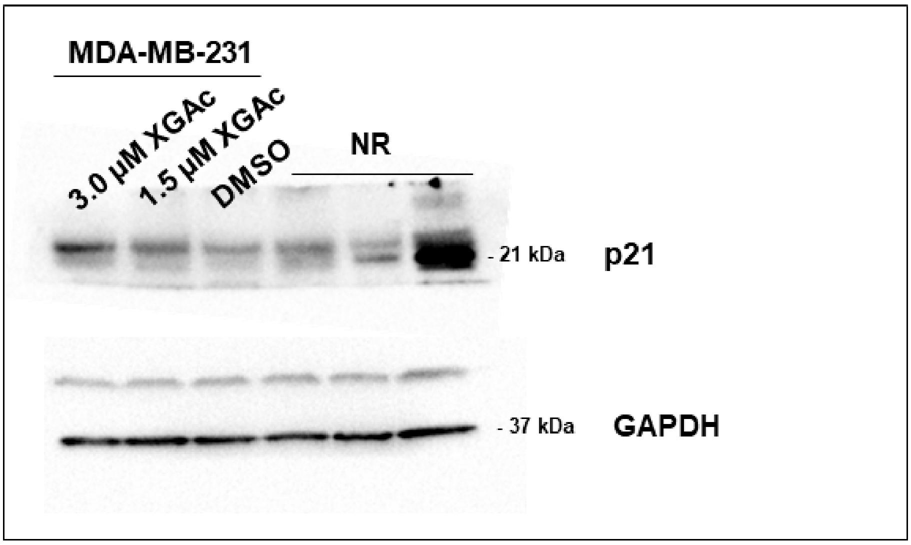
MDA-MB-231
3.0 µM XGAc
1.5 µM XGAc
DMSO
NR
- 21 kDa
p21
- 37 kDa
GAPDH

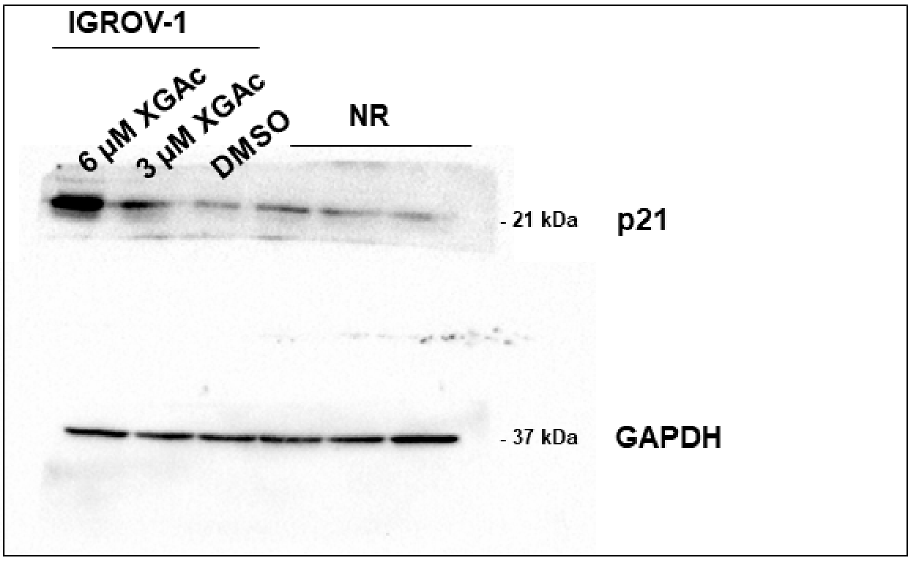
IGROV-1
6 µM XGAc
3 µM XGAc
DMSO
NR
- 21 kDa
p21
- 37 kDa
GAPDH

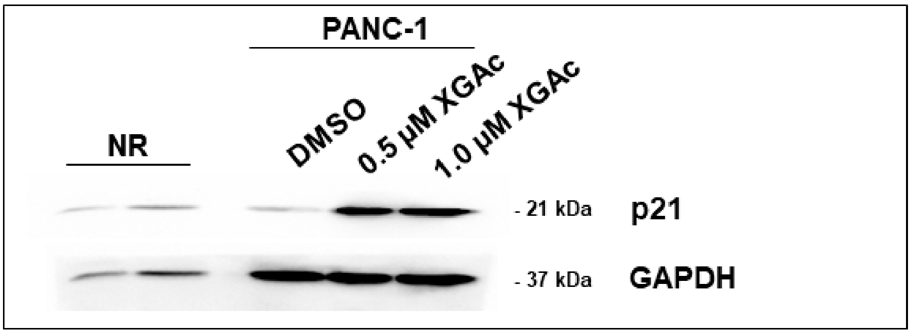
PANC-1
NR
DMSO
0.5 µM XGAc
1.0 µM XGAc
- 21 kDa
p21
- 37 kDa
GAPDH
